# Puberty in girls with Prader-Willi syndrome: cohort evaluation and clinical recommendations in a Latin American tertiary center

**DOI:** 10.3389/fendo.2024.1403470

**Published:** 2024-06-20

**Authors:** Caroline Gouveia Buff Passone, Luciana Felipe Ferrer Aragão, Ruth Rocha Franco, Junia Ellen Simioni Leite, Michelle Antonella Benitez Gonzalez, Priscila Schuindt de Albuquerque Schil, Marina Ybarra, Durval Damiani, Gerthe Femke Kerkhof, Renan Magalhães Montenegro Junior, Clovis Artur Silva

**Affiliations:** ^1^ Instituto da Criança e do Adolescente, University of Sao Paulo - Pediatric Endocrinology Department, Sao Paulo, Brazil; ^2^ Research Unit, Walter Candido University Hospital, Federal University o Ceara/Ebserh, Fortaleza, Brazil; ^3^ Children’s Hospital – London Health Science Center – Western University, London, ON, Canada; ^4^ Department of Endocrinology, Erasmus Medical Center (MC), Rotterdam, Netherlands

**Keywords:** Prader-Willi syndrome, hypogonadism, primary hypogonadism, secondary hypogonadism, hormone replacement therapy, puberty, gonadal disorders

## Abstract

**Introduction:**

Prader-Willi syndrome (PWS) is a genetic disorder characterized by hypothalamic-pituitary deficiencies including hypogonadism. In girls with PWS, hypogonadism can present early in childhood, leading to genital hypoplasia, delayed puberty, incomplete pubertal development, and infertility. In contrast, girls can present with premature activation of the adrenal axis leading to early pubarche and advanced bone age. We aim to evaluate the progression of puberty and adrenarche signals in girls with PWS.

**Methodology:**

A longitudinal retrospective cohort study included girls with PWS followed at a Pediatric Endocrinology Outpatient Clinic in a Tertiary University Hospital in Sao Paulo, Brazil from 2002 to 2022. Data collected via chart review included clinical information on birth history, breast and pubic hair Tanner stages, presence of genital hypoplasia, age at menarche, regularity of menstrual cycles, body mass index (BMI) z-score, final height, age of initiation of estrogen replacement and growth hormone replacement, as well as results for PWS genetic subtype; biochemical investigation (LH, FSH, estradiol, DHEA-S); radiographic bone age and pelvic ultrasound.

**Results:**

A total of 69 girls were included in the study and the mean age of puberty onset was 10.2 years in those who started puberty after the age of 8 years. Breast Tanner stage IV was reached by 29.1% girls at a mean age of 14.9 years. Spontaneous menarche was present in 13.8% and only one patient had regular menstrual cycles. Early adrenarche was seen in 40.4% of cases.

**Conclusion:**

Our study demonstrated in a large sample that girls with PWS often present with delayed onset of puberty despite frequent premature adrenarche. Based on our results, we suggest an estrogen replacement protocol for girls with PWS to be started at the chronological age or bone age of 12–13 years, taking into consideration the uterus size. Further prospective studies are needed.

## Introduction

Prader-Willi syndrome (PWS) is a genetic disorder resulted from the loss of paternal alleles in the 15.11q3 region (1). Genetic abnormalities include paternal deletion (60–75%), maternal uniparental disomy 15 (mUPD, 20–35%), imprinting center defect (ICD, 1–4%) and paternal chromosomal translocation (0.1%) (1, 2). PWS is characterized by neonatal hypotonia, early-onset obesity, neurodevelopmental delay, behavioral issues and hypothalamic-pituitary deficiencies, including hypogonadism (1, 3). Hypogonadism’s etiology and manifestation may exhibit variability among affected individuals (1, 2). In girls with PWS, hypogonadism can present early in childhood, leading to genital hypoplasia, followed by delayed puberty, incomplete pubertal development, and infertility (3). The presence of hypogonadism may lead to altered body composition, muscle weakness, poor body image, and reduced bone density, with limited data on its impact on cognition (4).

The etiology of hypogonadism in girls with PWS can be explained by a combination of primary gonadal insufficiency and hypothalamic dysfunction (5–9). The deletion of the SNORD 116 gene is associated with decreased transcription factors linked to the production of the enzyme proconvertase 1. The proconvertase 1 is responsible to convert pro-gonadotrophin releasing hormone (GnRH) into GnRH. Consequently, girls with PWS have reduced GnRH and subsequently diminished luteinizing hormone (LH) and follicle-stimulating hormone (FSH) pulses (10, 11). It has been reported that some girls may experience precocious puberty (1–3). In contrast to hypogonadism and with uncertain etiology, premature pubarche is also seen in girls with PWS (12).

Puberty is a dynamic process influenced by environmental and genetic factors Physiologically, puberty in girls typically commences between the ages of 8 and 13 years [13.14,15]. In the majority of cases, pubertal development in girls initiates with thelarche, followed by pubarche, and, lastly, menarche (13).. Traditionally, menarche occurs, on average, 2.3 years after the onset of thelarche (13, 14). Hormone replacement with estrogen, usually starting between 11- and 13-year-old, is the recommended therapy for female pediatric patients with hypogonadism in many syndromic scenarios other than PWS (e.g.: Turner Syndrome, Kallmann Syndrome, central tumors, chemotherapy) (15, 16). Recently, a study based on a retrospective Dutch cohort suggested estrogen replacement therapy as the clinical recommendations for adult women with PWS and hypogonadism (17). Currently, there are no recommendations regarding hormone replacement therapy in pediatric female patients with PWS and hypogonadism.

Most studies that looked at puberty and adrenarche in girls with PWS are limited to case reports or case series, predominantly described in European, Israeli and North American populations (8, 9, 12, 18). Our study looked at a large cohort of girls with PWS in a Tertiary University Hospital in Brazil. Our objective was to assess, describe and explore potential associations between clinical, biochemical, and imaging characteristics regarding puberty progression in girls with PWS. We then suggest a hormone replacement protocol tailored to female pediatric patients with PWS and hypogonadism.

## Methodology

Our study included female pediatric patients with PWS followed at the University of São Paulo Pediatric Outpatient Clinic from February 2002 to December 2022. Longitudinal retrospective data collected through chart review included clinical information on birth weight and length, breast and pubic hair Tanner stages (including age, bone age, and body mass index [BMI] z-score relative to these stages), presence of genital hypoplasia, age at menarche, regularity of menstrual cycles, body mass index (BMI) z-score, final height, age of initiation of estrogen replacement and growth hormone replacement, as well as results for PWM genetic subtype; biochemical investigation (LH, FSH, estradiol, dehydroepiandrosterone sulfate (DHEA-S)); radiographic bone age and pelvic ultrasound.

Patients were seen at least twice a year. Pubertal stage based on the Tanner classification (14) was evaluated by a pediatric endocrinologist at each visit (Breast- B1 to B4 and pubic hair P1 to P4). Standing height was measured using a *Harpenden* stadiometer, and weight was assessed on an accurate scale. Standard deviation scores (SDS) for height and BMI were computed according to World Health Organization (WHO) values (19).

Blood samples were all performed by the same laboratory at our center and included serum levels of LH, FSH, estradiol (immunochemiluminescence method), and DHEA-S (chemiluminescence method). Radiographic bone age was determined using the *Greulich and Pyle Atlas* and pelvic ultrasound was performed via supra-pubic by the same radiology team in our center.

Statistical analysis was performed using GraphPad Prism. Continuous variables were presented as median (maximum and minimum value) or mean ± standard deviation, as appropriated. Categorical variables were presented as frequency and percentage. For categorical variables, differences were calculated using Fishers exact test or chi-square test; for no categorical variables evaluated during the follow-up ANOVA; p-values < 0.05 were considered statistically significant.

## Results

A total of 72 female pediatric patients with PWS were selected through chart review. Three patients were excluded due to missing data. The remaining 69 patients were included in the study (**Table 1**). Of those 18 were pre-pubertal with an age younger than 7 years. The distribution of the different types of genetic mutation were described in **Table 1**. In 14% of cases, the DNA methylation test at the SNURF- SNRPN locus confirmed the syndrome, as this test detect 99% of cases with high specificity and sensitivity but cannot differentiate genetic subtypes.

**Table 1:** Baseline characteristics from a cohort of female pediatric patients with Prader-Willi Syndrome followed at a Pediatric Endocrinology Outpatient Clinic in a Tertiary University Hospital in Brazil from 2002 to 2022.

Characteristics	Total (N=69)
**Age**, years (mean ± SD) (minimum-maximum)	11.1 ± 5.6 1.6 - 25.6
**Follow-up,** years (mean ± SD)	7.0 ± 4.3
**Genetic subtype** N (%) Deletion UPD Imprinting center defect No data	27 (49.0) 25 (45.4) 3 (5.4) 14 (20.2)
**Birth Weight,** g (mean ± SD) **Birth Length,** cm (mean ± SD)	2450.0 ± 513.6 46.2 ± 3.0
**rhGH regular use** N, (%)	39 (56.0)
Genital hypoplasia* N/total (%) hypoplasia of labia minora clitoris hypoplasia labia minora synechie	21/31 (67.7) 18 (58.1) 5 (16.1) 3 (9.7)

UPD, uniparental disomy; rhGH, recombinant human growth hormone.

**Figure 1:**
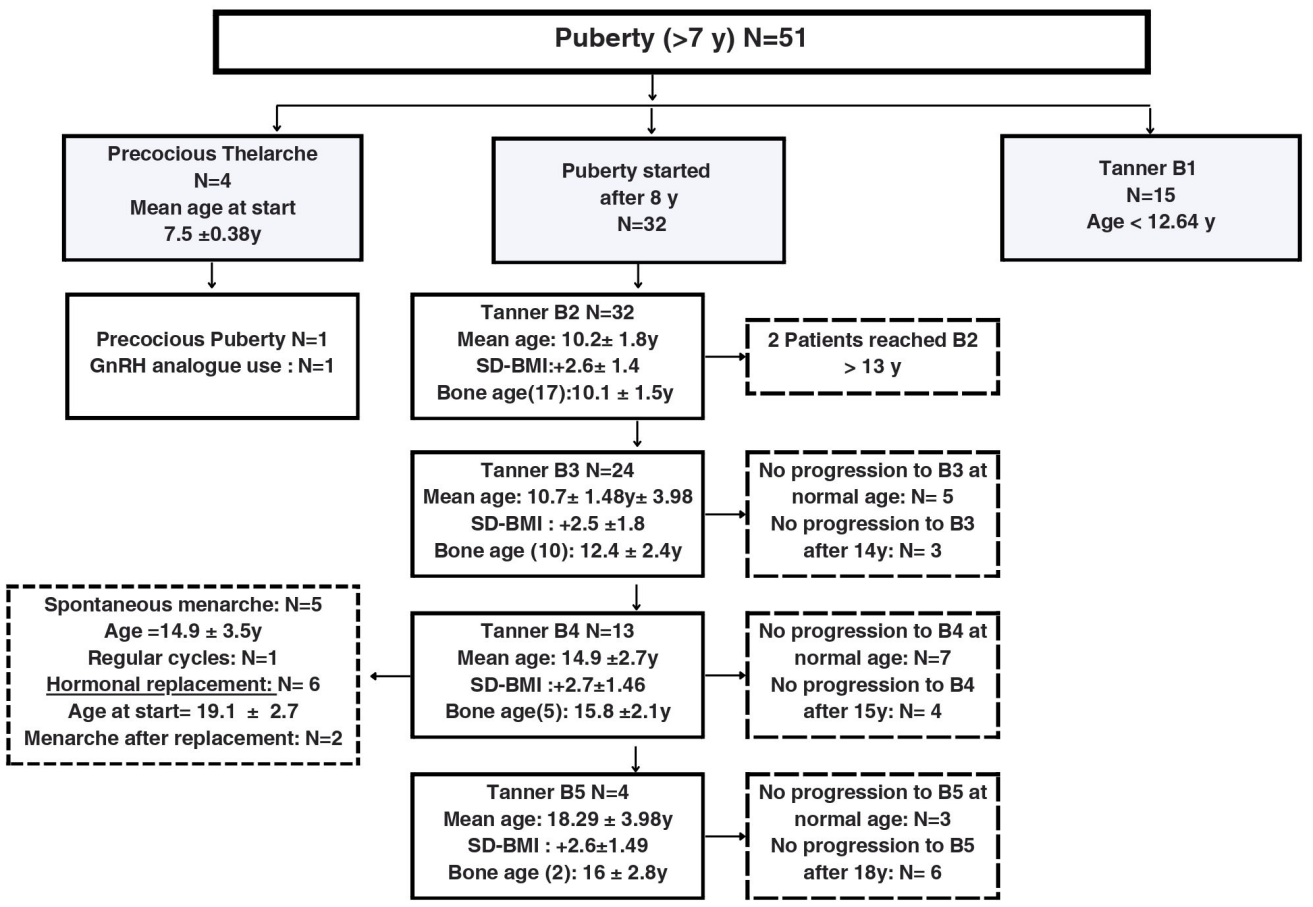
Description of the development of pubertal characteristics and breast Tanner stages in a cohort of 51 female pediatric patients older than 7 years old with Prader-Willi Syndrome followed at a Pediatric Endocrinology Outpatient Clinic in a Tertiary University Hospital in Brazil from 2002 to 2022. B, Breast Tanner Stage; y, years; SD-BMI, Standard Deviation Body Mass Index.

**Figure 2:**
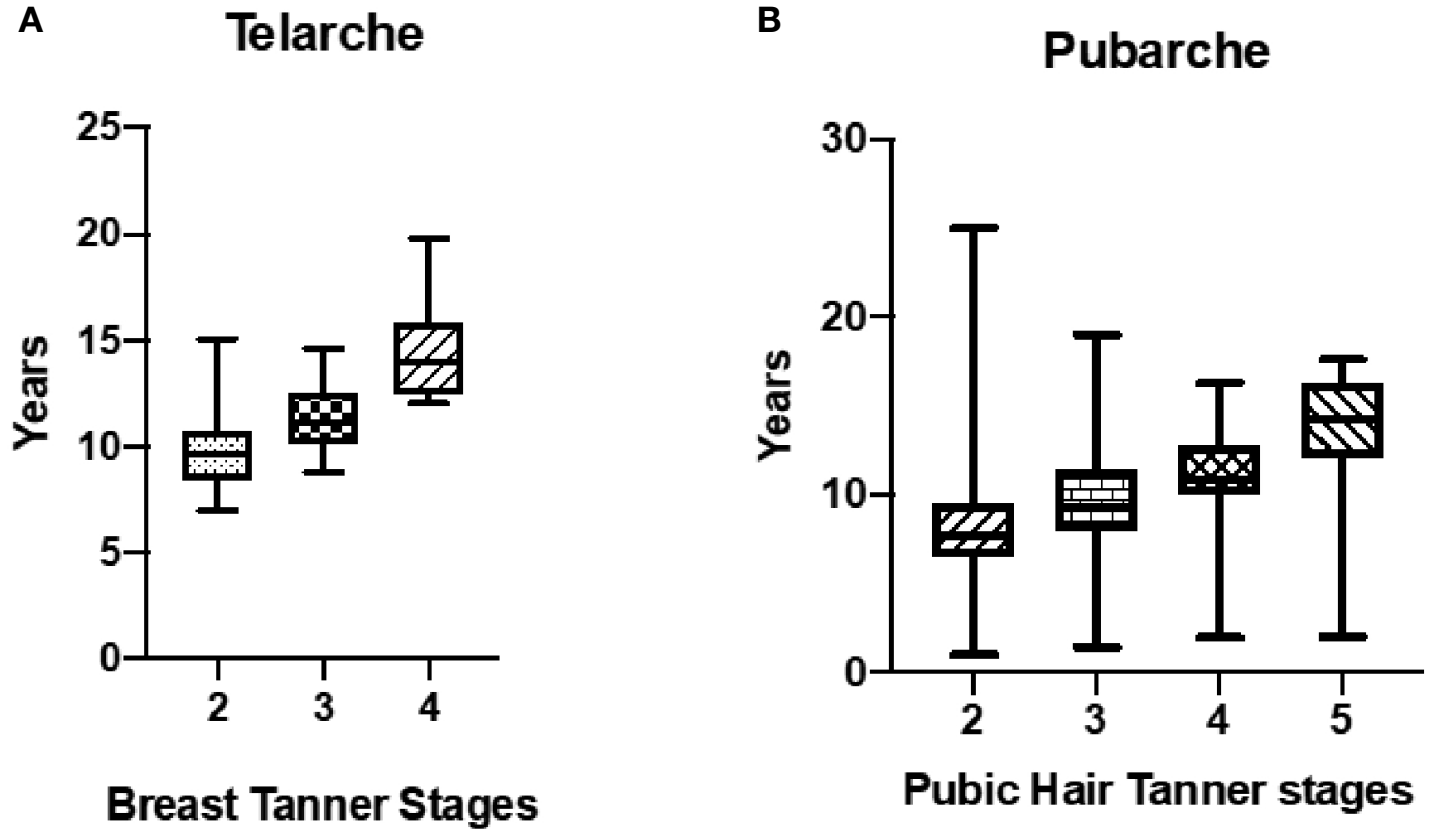
Age at breast development (**A**, n=51) and pubic hair development (**B**, n=42) in a cohort of female pediatric patients with Prader-Willi Syndrome followed at a Pediatric Endocrinology Outpatient Clinic in a Tertiary University Hospital in Brazil from 2002 to 2022.

From the fifty-one girls older than 7 years, fifteen were at tanner stage B1, 4 cases were diagnosed with precocious thelarche between age 7 and 8, with one case of central precocious puberty confirmed with a gonadotropin stimulation test and treated with GnRH analogue. Thirty-two patients started puberty at a mean age of 10.2 ± 1.8years and after around 4.7 years progressed to Tanner B4. Only 5 progressed to spontaneous menarche. Tanner stages, chronological and bone ages and clinical diagnoses of puberty are illustrated in **Figures 1**, **2A**.

**Figure 3:**
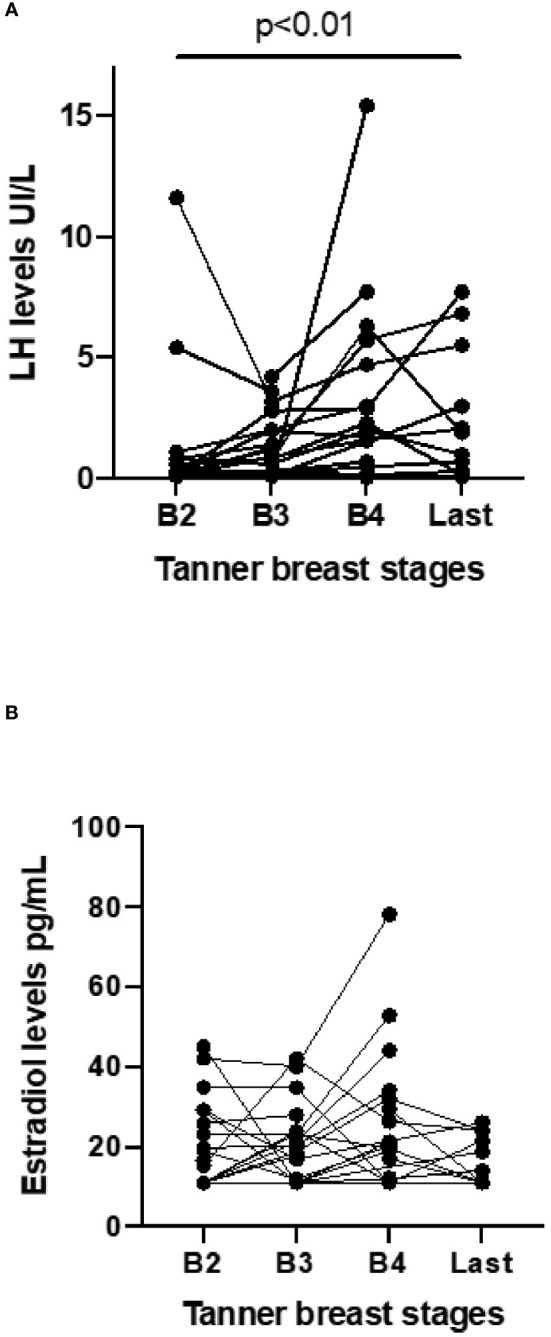
LH and estradiol levels according to breast development and at the last evaluation in female pediatric patients with Prader-Willi Syndrome followed at a Pediatric Endocrinology Outpatient Clinic in a Tertiary University Hospital in Brazil from 2002 to 2022 (n=34). LH levels: minimum detected level 0.1UI/L, estradiol levels: minimum detected level 11pg/ml **(A)** Significant difference in LH between Tanner stages (p<0.01, ANOVA) **(B)** No difference in estradiol between Tanner stages (p=0.10).

Girls with PWS reached puberty levels of LH and estradiol at a mean age of 11.8 ± 2.9 years and 11.6 ± 3.2 years, respectively (n=34, missed data=2). Nine patients exhibited basal stimulated LH (mean value: 1.9 ± 1.3 UI/L) one year after thelarche (Tanner B2) and 12 patients showed higher LH levels (mean value: 2.5 ± 1.7 UI/L) two years after reaching Tanner B2 (**Figure 3**). No patient had FSH above 15 UI/L. Although LH increased during Tanner stages, estradiol levels remained low (**Figure 3**).

At 15 years of age, 29.1% (n=7/24) of girls with PWS achieved Tanner B4, and 5 patients presented spontaneous menarche. Among those, uterus volume estimated via supra pubic ultrasound ranged between 10 and 17 cc, except for one case who presented with a volume of 28 cc. This patient was the only one who had regular menstrual cycles.

**Figure 4:**
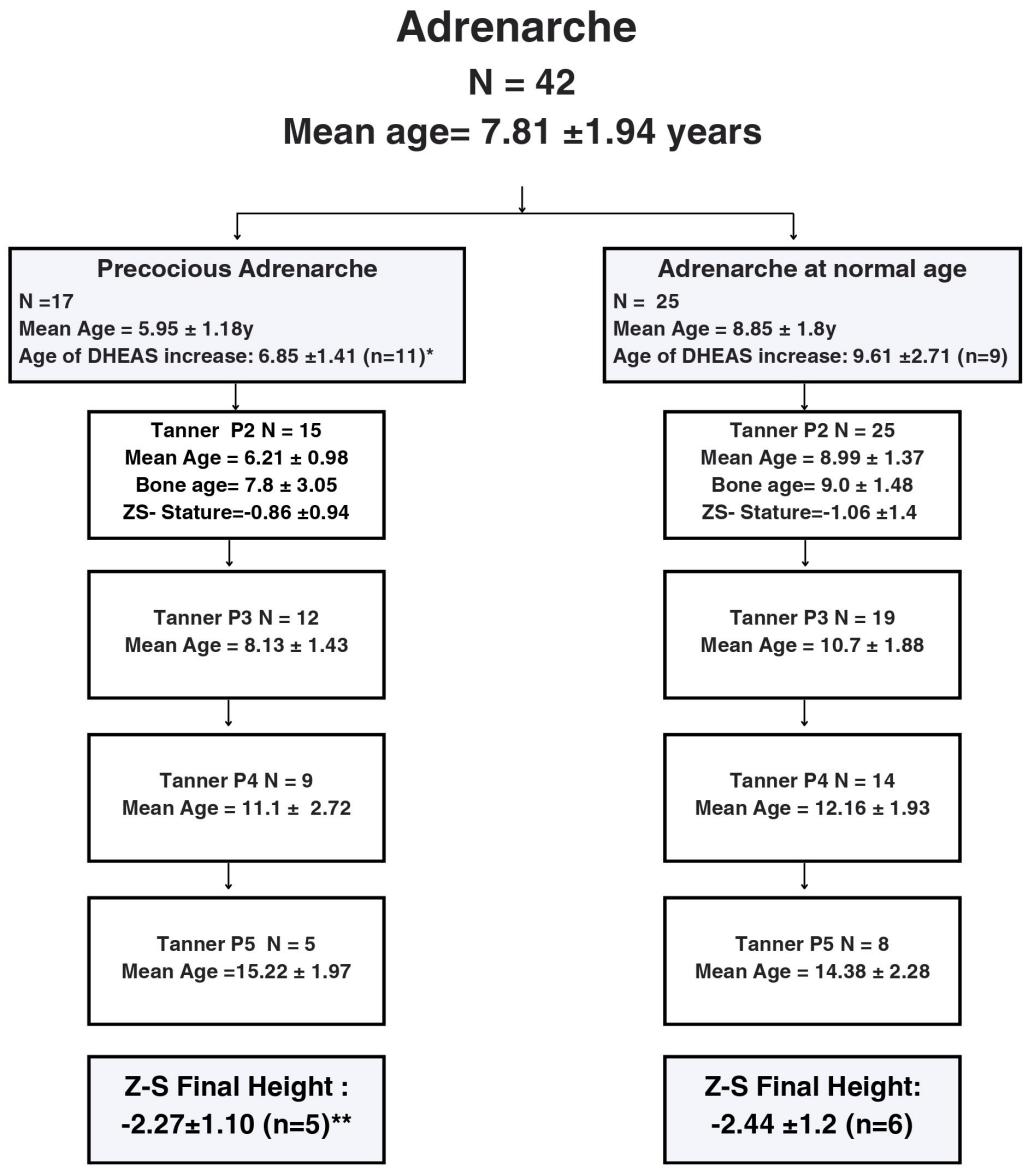
Flow-chart describing pubarche development according to pubic hair Tanner stages in a cohort of 42 female pediatric patients with Prader-Willi Syndrome followed at a Pediatric Endocrinology Outpatient Clinic in a Tertiary University Hospital in Brazil from 2002 to 2022. Abbreviations: P: pubic hair, y: years; DHEA-S: dehydroepiandrosterone sulfate.*Significant difference between age of DHEA-S increase (p=0.04, Chi-square). **No difference between height (Chi-square). Precocious adrenarche =start of adrenarche before the age of 7.

Data related to pubarche and adrenarche was evaluated in 42 of the 69 patients since 27 had no adrenarche. From these, 17 presented with early adrenarche. Mean DHEA-S levels at the onset of precocious adrenarche were 664.68 ng/mL (reference value: < 194ng/mL), with a significant difference in the age of DHEA-S increase (p=0.04). The mean z-BMI was 1.77 ± 1.44 SD at the commencement of precocious adrenarche. No associations were found between premature pubarche/adrenarche and final height (**Figures 2B**, **4**).

## Discussion

This longitudinal retrospective cohort study revealed a significant delay in pubertal progression in female pediatric patients with PWS. Major strengths of this article were the large sample size of 69 patients. Length of the study encompassed more than 20 years and the fact that it was conducted at the largest tertiary center in Latin America that has a dedicated multi-disciplinary and multiprofessional PWS clinic. Premature adrenarche was frequent, but its evolution and impact seemed to be limited, and the puberty development was impaired. As a response, we suggest a pubertal induction protocol for PWS girls in our service, emphasizing the potential benefits of hormone replacement therapy.

The onset of puberty in our sample was aligned with other studies (8, 9), occurring at a typical age of 10.2 years, with 91.3% of patients reaching breast Tanner stage B3 by the age of 13 years. We found a significant delay in pubertal progression (4.7 years between Tanner stage B2 and B4), with almost 30% of patients never reaching breast Tanner stage B4 by the age of 15 years. A study in Brazil, involving 665 healthy girls, reported that thelarche, pubarche, and menarche occurred at a mean age of 9.8, 10.2, and 11.7 years, respectively (13). It’s been reported that although most girls with PWS experience spontaneous breast development around the age of 12.6 years, most of them slowly progress through the pubertal stages and are usually delayed in reaching breast Tanner B5 or complete pubertal development (8, 9). We also saw a delayed mean age of LH and estradiol elevation which was not in alignment with Tanner stages.

In our study, only 5 girls experienced spontaneous menarche, at a mean age of 14.9 years. Those girls also experience irregular cycles and small uterus size Previous studies reported that only 8 to 44% of girls with PWS present with spontaneous menarche (6, 8, 9, 11, 12), and the majority develop irregular menstrual cycles (e.g.: oligomenorrhea, periodic spotting, and secondary amenorrhea) (9, 12). The mean age of menarche reported by those studies varied between 14, 17 and 20 years (8, 9, 12). We speculate that a proportion of the vaginal bleeding considered in previous studies as spontaneous menarche in girls with PWS may not indicate normal ovarian function or true menarche. Instead, it could be a result of the uterine endometrium estrogenization caused by the estrogen synthesized through the peripheral conversion of adrenal androgens (9).

The prevalence of hypogonadism in girls with PWS ranges from 54% to 100%, with the majority of studies indicating a prevalence higher than 80% (1–4, 9). The etiology of the hypogonadism reported in girls with PWS was secondary (central) in 53.8%, primary (gonadal failure) in 7.7% and a combination of both primary and secondary in 38.4% (6, 17, 18, 20, 21). Genital abnormalities, an indirect sign of hypogonadism, were seen in 67,7% of our sample. An Italian study with 42 girls with PWS reported genital abnormalities in 70% of cases (9).

Our study had one patient diagnosed with central precocious puberty. Previous studies also reported the presence of central precocious puberty, characterized by advancing bone age and increased growth velocity, although not always leading to early menarche (8, 9, 22–24). One case was treated with a GnRH analogue (9). The presence of mutations in the MKRN3, an imprinted gene located in the critical region of PWS on chromosome 15, was associated with familial precocious puberty and might explain it (25).

As expected, the onset of adrenarche occurred at a younger age (mean age of 7.81 ± 1.94 years), with the onset of early adrenarche occurring in 40.4% of the girls in our sample. Previous studies reported similar findings with a prevalence of early pubarche in girls with PWS ranging from 30% to 64% (12, 26),. Pubic hair development after reaching Tanner stage P2 occurred at a normal rate until stage P3, and then slowed down. This is comparable to other studies. Pubic hair progression in our study was less pronounced than in the Dutch group described by Siemensma et al., although the average ages reaching P3, P4, and P5 were comparable. Our study found that girls with PWS reached P2 and P3 at significantly younger ages compared to healthy Dutch children (12). Gaston and Stafford, analyzed 25 girls with PWS aged over 9 years and found that 64% of them had early pubarche, reaching P2 at a mean age of 7.3 years. Subsequent progress to P3 occurred at a mean age of 9.6 years, to P4 at 11.7 years, and to P5 at 16.8 years (26). These variations may be attributed to the diverse characteristics of the studied groups and multiple influencing factors on adrenarche in PWS, such as obesity, recombinant human growth hormone (rHGH) treatment, and environmental factors.

In our sample, girls with PWS with or without precocious puberty showed a similar bone age at Tanner stage P4. We speculate that the effects of adrenal androgens were likely temporary and had a limited impact on final height compared to the influence of puberty progression. Unfortunately, because of the young age of our study population, not all patients have yet attained final height, which might have impacted the results.

In our sample, girls who presented with premature adrenarche exhibited increased levels of DHEA-S at a younger age. It has been reported that in children with PWS, DHEA-S levels can be increased earlier, remaining elevated throughout childhood (26), but returning to normal levels in adulthood (8). Given the common occurrence of hypothalamic dysfunction in children with PWS, data suggest that increased DHEA-S levels may occur with less or even without any stimulation from the pituitary-adrenal-axis. The observation that pubarche progression was minimal after Tanner stage P4 for pubic hair may indicate that a normal pituitary-gonadal axis is crucial to reach Tanner stage P5.

Studies conducted in other syndromes with hypogonadism, such as Turner syndrome and central hypopituitarism, have demonstrated that estrogen replacement therapy contributes to a reduction in cardiovascular risk, improvement in bone mineral density, and cognitive enhancements, including motor speed as well as verbal and nonverbal processing time (15, 16, 27). Additionally, magnetic resonance imaging (MRI)-based investigations highlight the critical role of pubertal maturation and sex steroids in shaping brain structure, particularly during adolescence (28). It is evident that, while age can serve as a parameter for general developmental changes, pubertal maturation exerts unique and additive influences on structural neurodevelopmental trajectories. In patients with pre-existing hormone deficiencies of varying degrees, the inadequate myelination can potentially lead to premature aging and further impairment of the cerebral cortex and social performance (28). Moreover, in female adults with PWS there is a high prevalence of osteopenia (54%) and osteoporosis (14%), both important comorbidities related to estrogen deficiency (29).

We acknowledge that this is a retrospective observational study and it comes with its pitfalls. PWS is a rare genetic disease, making it difficult the development of prospective studies. The high prevalence of hypogonadism in our population led us to develop a pubertal induction protocol for girls with PWS in our service. Previous studies have shown the benefits of pubertal induction, encompassing multiple improvements in bone mineral density, body composition, psychological well-being, cognitive function, and cardiovascular aspects (15, 16, 28, 29). Hormone replacement therapy initiation may be delayed in patients with cognitive impairments due to factors like fear or lack of awareness of its benefits. However, it’s crucial to communicate and discuss the anticipated benefits with both patients and caregivers, highlighting the potential advantages of hormone replacement therapy.

**Figure 5:**
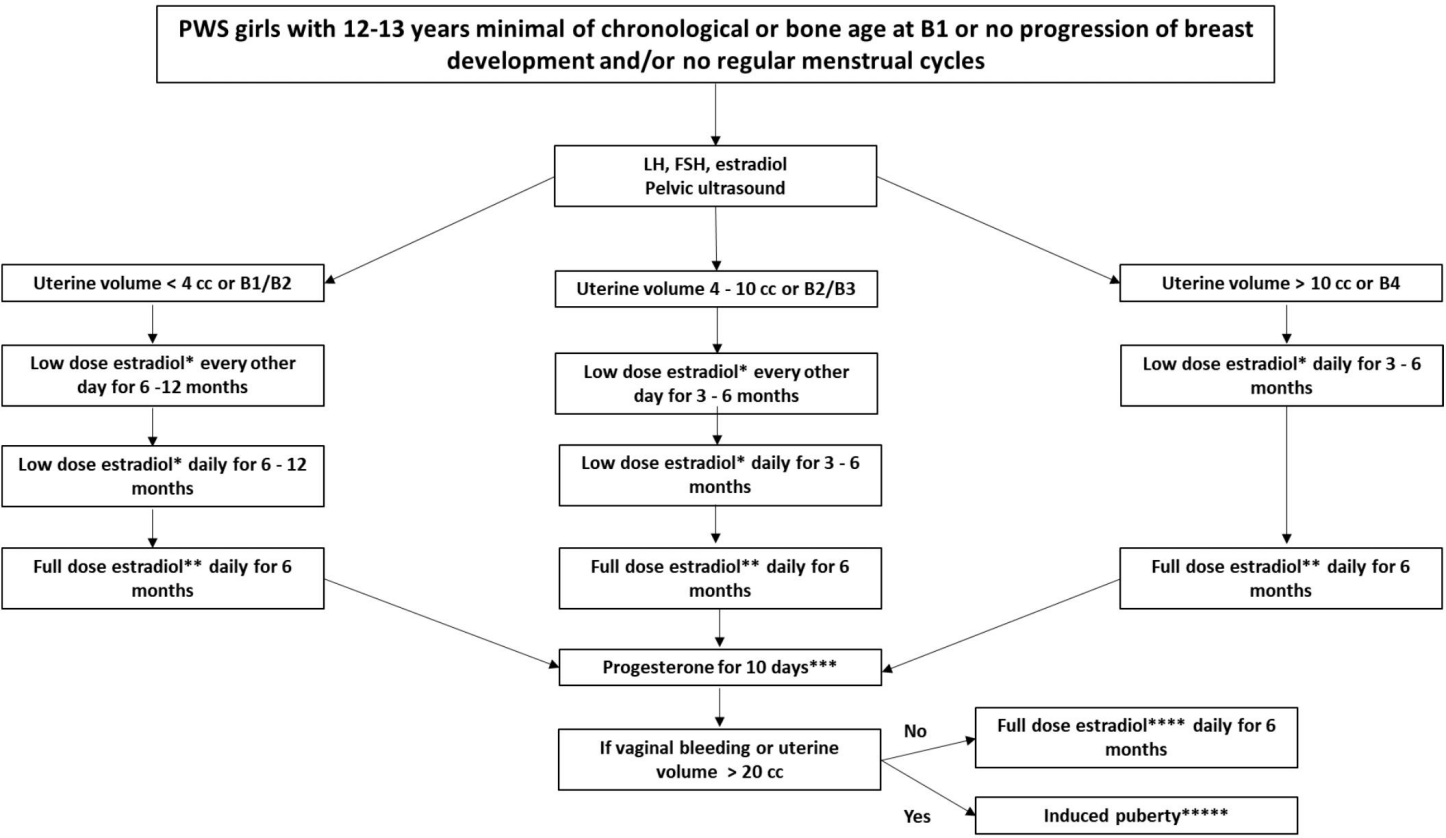
Recommended pubertal induction protocol for girls with Prader-Willi Syndrome. Abbreviation: PWS: Prader-Willi syndrome, B: breast. *17-beta-estradiol oral tablet: 0.5 mg or transdermal patch: 3.2mcg to 6.2mcg equivalent to one eighth to a quarter of patch overnight **17- beta-estradiol oral tablet: 1.0 mg or transdermal patch: 6.2mcg to 12.5mcg equivalent to one quarter to a half of patch overnight *** Micronized progesterone oral capsule: 100–200 mg. ****17- beta -estradiol oral tablet: 1.0–2.0 mg or transdermal patch 12.5mcg to 25mcg equivalent to a half or a full transdermal patch overnight. ^27^*****If puberty is induced, the maintenance phase can be carried out using full dose estradiol together with progesterone, or combined contraceptives continuously or intermittently, as desired by the patient and her caregivers.

We advocate for the treatment of hypogonadism in girls with PWS (**Figure 5**). Since the clinical (Tanner stages) and laboratory findings of puberty (LH, FSH, estradiol) may not be consistent and may differ between patients, we propose adopting the volume of the uterus seen in a supra-pubic ultrasound as an additional parameter for this protocol. The uterus size was based on pubertal stages and the normal uterus growth from childhood to adulthood (30, 31). Tanner stages could be difficult to be determined in children with obesity, especially when associate with a non-linear puberty progression. We recommend that the induction therapy with estrogen should start at the minimal age of 12–13 years of chronological or bone age, and breast Tanner stage I or no progression of breast development for more than one year or irregular menstrual cycles (15, 16, 27)..

For pubertal induction in girls with PWS, initial prescription of transdermal patches is typically recommended due to their adjustability through cutting and gradual dose escalation (15, 27). However, considering the prevalence of skin-picking behavior in PWS, we suggest commencing with low-dose of 17-b-estradiol oral tablets and titrating based on uterine size. Progesterone is then introduced for a 10-day period to assess vaginal bleeding. Subsequent maintenance of induced puberty involves the use of combined contraceptives continuously or intermittently, depending on patient and caregiver preferences.

Prior to treatment initiation, it’s crucial to assess thrombosis and breast cancer risk, potential drug interactions with hormonal replacement therapy, and to discuss hygiene, vaginal bleeding, behavioral changes, and possible weight gain with patients and caregivers (17). This emphasizes the necessity of follow-up with a multidisciplinary team, particularly for psychological support. Special attention is warranted for cases involving girls with PWS expressing sexual activity desires, necessitating heightened supervision to mitigate the risk of sexual abuse. This approach aims to tackle the specific challenges of hormonal replacement therapy in girls with PWS, fostering holistic care and enhancing health-related quality of life.

## Conclusion

Our study revealed a high prevalence of delayed puberty in girls with PWS, prompting the formulation of a recommended hormone replacement protocol for inducing puberty at a physiological age. Further prospective and multicenter studies are needed to validate these findings and refine treatment strategies.

## Data availability statement

The raw data supporting the conclusions of this article will be made available by the authors, without undue reservation.

## Ethics statement

The studies involving humans were approved by Instituto da Criança- HCFMUSP - Plataforma Brasil. The studies were conducted in accordance with the local legislation and institutional requirements. Written informed consent for participation in this study was provided by the participants’ legal guardians/next of kin.

## Author contributions

CP: Conceptualization, Data curation, Formal analysis, Funding acquisition, Investigation, Methodology, Project administration, Resources, Software, Supervision, Validation, Visualization, Writing – original draft, Writing – review & editing. LA: Conceptualization, Data curation, Formal analysis, Funding acquisition, Investigation, Methodology, Project administration, Resources, Software, Supervision, Validation, Visualization, Writing – original draft, Writing – review & editing. RF: Conceptualization, Formal analysis, Methodology, Project administration, Supervision, Validation, Writing – original draft, Writing – review & editing. JL: Data curation, Formal analysis, Investigation, Methodology, Project administration, Writing – review & editing. MG: Conceptualization, Data curation, Formal analysis, Investigation, Methodology, Project administration, Writing – original draft. PS: Data curation, Investigation, Methodology, Project administration, Writing – original draft. MY: Writing – review & editing. DD: Supervision, Validation, Visualization, Writing – review & editing. GK: Supervision, Validation, Visualization, Writing – review & editing. RM: Investigation, Supervision, Validation, Visualization, Writing – review & editing. CS: Resources, Supervision, Validation, Visualization, Writing – review & editing.
